# Will future maize improvement programs leverage the canopy light-interception, photosynthetic, and biomass capacities of traditional accessions?

**DOI:** 10.7717/peerj.15233

**Published:** 2023-04-27

**Authors:** Ahamadeen Nagoor Mohamed Mubarak, Mohammathu Musthapha Mufeeth Mohammathu, Arachchi Devayalage Nishantha Thissa Kumara

**Affiliations:** Department of Biosystems Technology, Faculty of Technology, South Eastern University of Sri Lanka, Oluvil, Sri Lanka

**Keywords:** Chlorophyll content, Fractional interception, Leaf area index, Light extinction coefficient, Photosynthetic rates, Biomass, Grain yield, Radiation use efficiency, Canopy architecture, Water use efficiency

## Abstract

Maize germplasm has greater latent potential to address the global food and feed crisis because of its high radiation, water and nutrient efficiencies. Photosynthetic and canopy architectural traits in maize are important in determining yield. The present study aimed to screen a subset of local maize accessions in Sri Lanka to evaluate their photosynthetic, biomass and yield related traits and to identify resource efficient germplasm. Experiments were carried out in the Ampara district of Sri Lanka. Eight maize accessions *viz*; *SEU2, SEU6, SEU9, SEU10, SEU14, SEU15, SEU17* and *SEU17* and two elite F_1_ cultivars (*cv*. *Pacific-999* and *cv. Bhadra*) were analyzed under field conditions. Our results showed that maize genotypes produced a lower leaf area index (LAI) at the third and tenth week after field planting (WAP). However, the LAI was significantly increased in six WAP by *Pacific-999*, *SEU2, SEU9*, and *SEU15*. A similar trend was observed for percentage of light interception at three WAP (47%), six WAP (>64%), and decreased at 10 WAP. In addition, LAI maximum values were between 3.0 and 3.5, allowing 80% of the incident light to be intercepted by maize canopies. The estimated light extinction coefficient (*k*) remained lower (<0.5), suggesting that maize leaves are eractophilic canopies. Although fractional interception (*f*) varies, *SEU2* and *SEU9* had the highest values (0.57), and quantum yields of PSII (>0.73) in dark-adapted leaves. In addition, *Pacific-999*, *SEU2*, *SEU9*, and *SEU17* had significantly higher rates of photosynthesis with minimal stomatal conductance and transpiration rates. As a result, they outperformed the control plants in terms of biomass, cob weight and grain yield. This suggests that native maize germplasm could be introduced as novel, less resource-intensive cultivars to sustain global food security.

## Introduction

Maize (*Zea mays*, L.) is a major grain-producing cereal throughout the world. Maize also serves as a dual-purpose crop with multiple end-products that are used for household consumption and residues for livestock (Lenné & Wood, 2016). Effective plant screening is one of the key strategies used by breeders and agronomists to create superior cultivars promising improved biomass and able to withstand challenging environments (Avramova et al., 2016). Therefore, screening and identification of elite germplasm necessitates the understanding of the various plant processes involved in its eco-physiological responses. A crop cultivar is defined as a well developed crop coming from old population that has been tailored to the regional climate and adapted to farmer agronomic practices (Palumbo et al., 2017). According to recent evidence shows that over 28,000 unique seed collections of maize genetic diversity have been identified (O’Leary, 2016), while Sri Lanka has roughly 697 maize germplasm, of which 35 are local maize accessions (Plant Genetic Resources for Food & Agriculture, 2008). Preliminary studies reported such Sri Lankan maize accessions exhibit diverse nature in terms of morphology, canopy architecture and biomass (Mufeeth et al., 2020).

Photosynthetic properties in crops are vital not only for CO_2_ abatement but also to tackle rising food demands (Guidi, Lo Piccolo & Landi, 2019). Numerous attempts have been reported in selecting photosynthetically efficient plants among maize populations (Baghel, Kataria & Jain, 2019; Nasar et al., 2021) and other cereal crops such as rice (Feldman et al., 2017; Nagoor, 2013), wheat (Gaju et al., 2016) and barley (Sadura et al., 2019). Crop production is the result of a series of physiological events that begin with CO_2_ diffusion through the stomata into the intercellular spaces, followed by CO_2_ assimilation (Taiz et al., 2015). Examining key photosynthetic parameters, researchers can explore how biochemical and biophysical components of photosynthesis influence net carbon assimilation in response to environmental changes, natural phenotypic variances, genetic mutations and genetic modification through biotechnological means. Towards this direction, the widely employed and robust technique of chlorophyll fluorescent measurement aids in the assessment of both photosystems functionality giving information in energy transfer and photochemistry efficiency (Murchie & Lawson, 2013). Such techniques have been widely employed in a variety of wheat genotypes (Ajigboye et al., 2017).

The canopy architecture of crops exert control in solar radiation absorption, and attenuation which eventually contributes to the final yield (Azam-Ali & Squire, 2002). In this regard, the primary interface for mass and energy exchange between the atmosphere and plants is the leaves, which establish the majority of the canopy surface of field crops. The surface area of leaves is directly proportional to important processes such as assimilation rates, canopy light interception, respiration, transpiration, (Pokovai & Fodor, 2019) and carbohydrate metabolism (Murchie, Pinto & Horton, 2009). As such, the LAI is a key structural characteristic of the plant canopy, described as the ratio between the one-sided leaf surface area of the entire canopy with the corresponding ground area underneath it (Rahman et al., 2010). Both farmers and plant scientists use this measure to evaluate plant development. Crop modelers can also use it to scale up processes at the leaf and plant level to investigate physiological activities (Novelli et al., 2019). The canopy architecture, photosynthetic characteristics, and yield potentials of Sri Lankan maize accessions have not been investigated. Therefore, it is necessary to unravel such latent potential of native accessions to sustain maize production in Sri Lanka and elsewhere.

In this context, our present study aims to identify the photosynthetic and biomass attributes of the local maize accessions investigating the possibility of finding superior candidates to serve as novel genomic resources in future maize breeding programs. This could promote the development of less resource-intensive cultivars to sustain global food security.

## Materials and Methods

### Experimental location

Plant screening trials were carried out at the Agro Tech Park, Malwatta (7°20′N and 81°44′E, altitude 16.0 m above sea level), in the Ampara region of Sri Lanka. This experimental test location is categorized as a dry zone, with sandy loam soils. The average monthly rainfall and temperature readings are 127.1 mm and 30.3 °C, respectively (Mubarak et al., 2022).

### Plant material, growth conditions and experimental design

The experimental site was ploughed at the depth of 40–45 cm and levelled to facilitate soil physical properties that encourage maize plant root penetration (du Plessis, 2003). The field layout was designed with a randomized complete block design (RCBD), comprised of 10 treatments (10 maize germplasms) with four replicate (plots)/treatment (1.5 × 3.0 m^2^ ). Ten plants were grown in each treatment plot. The tested maize germplasm included *Pacific-999*, an imported F_1_ hybrid variety from Thailand, and eight high-performing local maize accession, which were designated as *SEU2*, *SEU6*, *SEU9*, *SEU10*, *SEU14*, *SEU15*, *SEU16*, and *SEU17*, and *cv.Bhadra* (control plant), a popular open pollinated recommended variety is released by the Department of Agriculture in Sri Lanka and was sowed at a spacing of 30 × 60 cm. Subsequently, the following standard agronomic practices were implemented as recommended by the Department of Agriculture, Sri Lanka, as follows. Seeds were directly sown into the field after being soaked for 24 h to encourage germination. A total of 1 week prior to planting, the basal fertilizer dressing was applied at the rates of 75 kg/ha of urea, 100 kg/ha of P_2_O_5_, and 50 kg/ha of K_2_O. Another 250 kg/ha of urea was used as a top dressing after the first 4 weeks of crop establishment, and then earthing up was done between crop rows to reduce weed development. The flooding irrigation method was used to provide the required amount of water throughout the cropping season.

### Plant growth and development measurements

Maize seed germination count was carried out 3 days after sowing and continued monitoring on daily basis until the first week of field planting to estimate germination percentage. The leaf count from randomly selected five plants per plot was recorded every week until the flag leaf of the plant emerged to calculate the average leaf development rate per week. Followed by the flag leaf development, the mean height of the crop stand was measured from five randomly selected plants from each plot and the measurement was taken from the soil surface to the flag leaf using 1 mm least count measuring tape. The mean days taken to 50% tassel emergence were recorded for each accession and compared. At the harvesting stage, the number of well-developed cobs per plant was counted. The plant physiological measurements were recorded at three distinct growth and developmental stages of maize accessions. At the early vegetative stage, (three weeks after field planting, three WAP) tasseling stage (six WAP) and grain filling stage (10 WAP).

### Photosynthetically active radiation and leaf area index measurements

A ceptometer (AccuPAR, LP-80; Meter Group Inc., Pullman, WA, USA) was used to measure photosynthetically active radiation (PAR). When the exterior PAR sensor measurement was greater than 600 μmols m^−1^ s^−1^, the device was calibrated in accordance with the manufacturer’s instructions. Thereafter, the PAR at the above crop canopy was measured with an external light sensor (Apogee SQ110; Apogee, Santa Monica, CA, USA), while the below PAR was measured by placing 5 cm above the ground with the 80 cm probe which contains 80 independent sensors, spaced 1 cm apart (LP- 80, AccuPAR; Meter Group Inc., Pullman, WA, USA). The photosensors measure photosynthetically active radiation in the 400 to 700 nm waveband by placing it 5 cm above the ground. To reduce the portion of diffuse radiation into the plant canopy, at least 8–10 measurements were taken per plot between 10.30 a.m. and 1.30 p.m. Subsequently, light interception percentage (LI %) per individual canopy was calculated at the early vegetative stage (three WAP), tasseling (six WAP) and grain filling (10 WAP) stages respectively. Thereafter, the LAI was estimated in the above stages using the leaf distribution parameter (χ) and the ratio of below and above PAR level (Francone et al., 2014). Regression lines were fitted with LAI verse LI % to find out the optimum amount of solar radiation that the crop canopy be achieved. Subsequently, the light extinction coefficients (*k*) for radiation of each maize accession were calculated by applying Eq. (1) as stated by Monsi & Saeki (1953) and Lambert-Beer law. Then the graphs were plotted against *ln* (*I/Io*) *vs* LAI, which specifies *k* and the crop canopy architecture (Azam-Ali & Squire, 2002).

(1) $$I/{I_o} = {e^{ - kLAI}}$$

where *I*_*o*_ and *I* are above and below canopy PAR values. Subsequently, the fraction of radiation intercepted by the crop canopy (*f*) was computed (Eq. (2)).

(2) $$f = 1 - {e^{( - kL)}}$$

### Leaf chlorophyll fluorescence parameters

A portable fluorometer (FluorPen FP 100; Photon Systems Instruments, Drasov, Czech Republic) was used to measure maximum quantum yield photosystem II (PSII) at light (*Qy*_*ligh*t_: Fv′/Fm′) and dark (*Qy*_*dark*_: Fv/Fm) adapted leaf from each treatment and control. The flash pulse of the blue light LED emitter (455 nm) with the maximum intensity was set to 3,000 µmol m^−2^s^−1^. The *Qy*_*ligh*t_ measurements were taken on the fully developed youngest leaves from randomly selected five plants. The same leaves were covered with aluminium foil for 30 min to take *Qy*_*dark*_ (Naidoo & Naidoo, 2018). All the indicated measurements were taken at the three, six and 10 WAP stages.

### Chlorophyll content

The leaf chlorophyll content in the SPAD unit was measured by employing SPAD 502 plus (Konica Minoltkoptics Inc., Wayne, NJ, USA). Initially, the instrument was calibrated then it was checked by a reading checker to ensure the calibration. The measurements were taken at three, six and 10 WAP stages. The average chlorophyll content measurement was taken from the base, middle, and tip of the youngest fully expanded leaves from randomly selected five plants per field plot.

### Leaf gas exchange parameters

Net CO_2_ assimilation rates (*A*_*N*_) (µmol CO_2_ m^−2^ s^−1^), transpiration rates (*E*) (mmol H_2_O m^−2^ s^−1^), stomatal conductance (*gs*) (mmol H_2_O m^−2^ s^−1^) were measured with an Infrared Gas Analyzer (LI-6800; LI-COR, Lincoln, NE, USA) portable photosynthesis system. Initially, machine calibration was performed including IRGA zeroing when the machine was attached with fresh Drierite^®^ and soda-lime. This procedure was precisely followed to avoid any temperature-induced zero shifts after acclimating to the chamber’s conditions (Ramazan et al., 2021). Before the spot leaf measurements, the warmup test was performed to check errors and faults that might undermine measurements. Then the following environmental conditions were maintained inside the chamber: leaf area of 6 cm^2^, leaf temperature at 30 °C, air flow rates at 600 µmol s^−1^, the relative air humidity within leaf chamber 70 ± 1%, CO_2_ concentration at reference IRGA line was set at 410 µmol mol^−1^, the light intensity was kept at 1,700 μmol mol^-1^ photons m^−2^ s^−1^ (10% blue and 90% red). On the 45^th^ days after field planting, the youngest fully expanded leaves from three plants per plot were measured. Then the leaf was clamped and the measurement was taken after 5 min to stabilize the photosynthetic parameters. All photosynthetic parameters were measured from 9.00 am to 12.00 noon on clear, cloudless days to ensure the plants are engaged active photosynthetic state while minimizing plant stress due to light acclimation.

### Above-ground dry mass and yield components

Once the plants had achieved their stage of physiological maturity, three randomly chosen plants from each plot were chosen and cut at the ground level. To acquire the above-ground biomass, the plant parts were divided into stem and cobs and oven-dried at 80 °C until completely dry (AGDM). Additionally, the fresh cobs were taken from four randomly chosen plants within each field plot in order to quantify the yield components and allow them to air dry so that the number of grains was recorded. At a moisture level of 12 percent, individual cob weight, and 100-kernels weight were measured.

### Statistical analysis

Univariate analysis of variance (ANOVA) was carried out to evaluate the significant (*p* < 0.05) variations in maize accessions in comparison to control (*cv.Bhadra*). Estimation of pairwise phenotypic correlation between physiological characteristics of maize germplasms were performed using SPSS (version 25.00) software.

## Results

### Plant growth and development parameters

The features of maize growth and development are shown in Table 1. Out of 10 germplasms, *SEU2* had the highest plant germination percentage (84%) followed by the control maize variety (83%). The leaf development rates were evaluated. Only one germplasm (*SEU17*) produced considerably higher growth rates (two leaves/week^−1^) than that of the control, while the other assessed germplasm had roughly identical leaf development rates (1.73 week^−1^). In comparison to the other examined germplasm, the *SEU2* had displayed the earliest days required for producing 50% tassel (44 d). In contrast, *SEU17* showed delayed flowering characteristic (54 d) than that of control plants (46 d). In terms of plant height, *SEU17* exhibited a significantly taller plants (221 cm), closely followed by *SEU2* (201 cm), *SEU10*, and *SEU14* respectively than *cv. Bhadra* (165 cm).

**Table 1:** Variations in plant growth and developmental characteristics of maize accessions.

Maize germplasms	Germination percentage	Leaf development rate (No. of leaves per week)	No. of days for 50% tasseling	Average height (cm)
*SEU2*	84.00 ± 4.32	1.84 ± 0.08	44.25 ± 0.25	201.8 ± 6.5*
*SEU6*	74.00 ± 6.22	1.81 ± 0.02	48.50 ± 0.50	169.5 ± 3.9
*SEU9*	68.00 ± 7.83	1.84 ± 0.05	48.00 ± 1.58	176.9 ± 4.8
*SEU10*	79.00 ± 7.0	1.86 ± 0.10	50.50 ± 0.50	198.2 ± 3.0*
*SEU14*	50.00 ± 6.22*	1.89 ± 0.04	53.50 ± 0.87*	186.3 ± 5.4*
*SEU15*	81.00 ± 7.37	1.86 ± 0.06	46.75 ± 0.85	174.0 ± 4.6
*SEU16*	71.00 ± 4.73	1.71 ± 0.04	46.75 ± 0.48	174.2 ± 4.8
*SEU17*	73.00 ± 6.61	2.05 ± 0.09*	54.00 ± 2.61*	221.3 ± 5.7*
*Pacific-999*	83.00 ± 5.74	1.73 ± 0.06	45.75 ± 0.75	160.9 ± 3.6
*cv.Bhadra*	83.00 ± 3.00	1.73 ± 0.02	45.75 ± 0.25	165.3 ± 3.8
F_(9,39)_	5.19	2.66	9.27	6.15
*p*	0.001	0.02	0.001	0.001

**Note:**

* Indicates significant differences maize populations per treatment than *cv.Bhadra* (*p* < 0.05). The values correspond to the mean of each parameter ± SE (*n* = 20).

Each maize accession’s leaf area index (LAI) was compared. The control plants typically produce a lower LAI at 3 WAP (1.44), pronounced higher at six WAP (2.3), and a lower LAI at 10 WAP (1.93), therefore the mean LAI remained at 1.90 (Table 2). However, there were appreciable differences across the treatments. Out of all screened germplasms, *SEU2* produced mean LAI of 2.3 and significantly enhanced LAI at six WAP (3.2) and 10 WAP (2.3). The majority of the tested maize accessions showed a similar trend. The LAI produced by the *SEU9*, *SEU15*, and *Pacific-999* at 6 WAP was considerably higher than that of *cv.Bhadra*.

**Table 2:** Leaf area index of maize germplasm at different developmental stages.

Maize germplasms	LAI at 3 WAP	LAI at 6 WAP	LAI at 10 WAP	Overall mean LAI
*SEU2*	1.58 ± 0.10	3.02 ± 0.24*	2.31 ± 0.05*	2.30 ± 0.09*
*SEU6*	1.06 ± 0.06*	2.60 ± 0.11	1.82 ± 0.07	1.83 ± 0.05
*SEU9*	1.38 ± 0.09	2.98 ± 0.17*	1.61 ± 0.10*	2.02 ± 0.06
*SEU10*	1.28 ± 0.09	2.56 ± 0.15	2.32 ± 0.11*	2.10 ± 0.06
*SEU14*	0.94 ± 0.04*	2.39 ± 0.13	1.60 ± 0.12*	1.72 ± 0.06
*SEU15*	1.46 ± 0.08	2.93 ± 0.10*	2.10 ± 0.16	2.16 ± 0.06*
*SEU16*	1.33 ± 0.06	2.24 ± 0.14	2.35 ± 0.19*	1.94 ± 0.03
*SEU17*	1.52 ± 0.13	2.66 ± 0.12	2.15 ± 0.16	2.17 ± 0.08*
*Pacific-999*	0.98 ± 0.10*	3.03 ± 0.18	1.57 ± 0.15	1.86 ± 0.08
*cv.Bhadra*	1.44 ± 0.07	2.32 ± 0.13	1.93 ± 0.10	1.90 ± 0.05
F_(9,158)_	14.21	6.77	37.26	16.30
*p*	0.001	0.001	0.001	0.001

**Notes:**

LAI, Leaf area index; WAP, week after planting.

* Indicates significant differences between corresponding maize accession and cv.*Bhadra* respectively (*p* < 0.05). The values correspond to the mean of each parameter ± SE (*n* = 20).

Comparison of pattern reception of photosynthetically active radiation (PAR) and subsequent light attenuation properties in the canopies, at three WAP, six WAP and 10 WAP indicated that the *SEU2* showed increasing percentage, while the control variety showed a lower percentage in all stages (47%, 64% and 63% respectively). The magnitude of the LAI and light extinction characteristics may account for variation in light attenuation (Table 3).

**Table 3:** PAR values at the above and below canopy positions PAR amount of attenuation and percentage at different stages of maize plants.

Measuring stage	Maize germplasms	PAR levels (μmolm^−2^s^−1^ )		Amount of PAR attenuation	% of PAR attenuation
		Above canopy	Below canopy		
3 WAP	*SEU2*	1,773.04 ± 69.24	874.67 ± 47.15	898.37 ± 47.75	50.63 ± 0.02
	*SEU6*	1,702.81 ± 80.68	1,065.78 ± 62.66*	637.03 ± 32.84	37.85 ± 0.02*
	*SEU9*	1,706.89 ± 82.88	932.17 ± 81.18	774.72 ± 40.49	46.65 ± 0.03
	*SEU10*	1,817.15 ± 62.35	983.74 ± 45.09	833.41 ± 53.96	45.47 ± 0.02
	*SEU14*	1,699.15 ± 102.25	1,029.84 ± 70.13	589.99 ± 30.24*	36.80 ± 0.01*
	*SEU15*	1,688.46 ± 78.34	869.03 ± 66.07	819.43 ± 42.79	49.29 ± 0.02
	*SEU16*	1,891.14 ± 39.57	1,072.66 ± 30.97*	818.48 ± 37.48*	43.12 ± 0.01
	*SEU17*	1,841.64 ± 54.84	956.11 ± 57.53	885.53 ± 57.02	48.09 ± 0.03
	*Pacific-999*	1,728.26 ± 68.78	1,130.62 ± 54.05*	597.63 ± 52.66*	34.13 ± 0.02*
	*cv.Bhadra*	1,762.79 ± 61.26	919.21 ± 42.88	808.97 ± 53.20	47.30 ± 0.02
	F_(9,182)_	–	8.19	12.98	12.07
	*p*	–	0.0001	0.0001	0.0001
6 WAP	*SEU2*	1,831.43 ± 33.18	499.97 ± 43.39*	1,286.26 ± 64.68	71.58 ± 2.47
	*SEU6*	1,901.81 ± 18.18	589.93 ± 29.90	1,285.74 ± 40.53	67.59 ± 1.96
	*SEU9*	1,972.08 ± 20.44	573.19 ± 47.89	1,366.58 ± 42.33	70.65 ± 2.22
	*SEU10*	1,862.50 ± 62.17	648.50 ± 52.84	1,250.46 ± 46.02	65.26 ± 2.60
	*SEU14*	1,744.03 ± 59.94	605.74 ± 48.33	1,158.73 ± 29.03	65.25 ± 2.35*
	*SEU15*	1,893.05 ± 41.68	471.23 ± 21.65*	1,341.82 ± 28.33	73.10 ± 1.05
	*SEU16*	1,966.73 ± 32.13	785.65 ± 54.66	1,181.08 ± 40.93	60.42 ± 2.43
	*SEU17*	1,921.29 ± 11.17	526.46 ± 21.69	1,344.09 ± 31.79	72.21 ± 1.15
	*Pacific-999*	1,860.16 ± 14.47	509.18 ± 35.08*	1,328.38 ± 41.54	71.04 ± 2.40
	*cv.Bhadra*	1,909.05 ± 37.06	689.39 ± 51.29	1,219.67 ± 38.61	64.26 ± 2.24
	F_(9,182)_	–	6.45	3.45	4.68
	*p*	–	0.0001	0.001	0.0001
10 WAP	*SEU2*	2,250.65 ± 65.13	677.83 ± 68.60	1,572.83 ± 65.10	70.02 ± 2.79
	*SEU6*	2,087.17 ± 59.79	760.30 ± 59.99	1,247.99 ± 109.76	61.63 ± 3.27
	*SEU9*	1,663.30 ± 222.48	578.15 ± 83.00	1,085.15 ± 152.15*	63.52 ± 1.45
	*SEU10*	2,143.41 ± 48.80	652.10 ± 51.99	1,491.31 ± 43.98	69.71 ± 2.20
	*SEU14*	1,636.65 ± 223.39	600.78 ± 91.51	1,035.88 ± 144.30*	63.17 ± 1.90
	*SEU15*	1,799.23 ± 205.43	534.16 ± 69.11	1,265.06 ± 152.45	70.17 ± 1.90
	*SEU16*	2,136.79 ± 63.83	647.35 ± 22.45	1,512.20 ± 75.13	68.82 ± 2.64
	*SEU17*	1,728.60 ± 259.44	633.13 ± 80.67	1,095.48 ± 187.79	60.39 ± 3.16
	*Pacific-999*	1,728.60 ± 259.44	633.13 ± 80.67	1,095.48 ± 187.79	60.39 ± 3.16
	*cv.Bhadra*	2,134.06 ± 90.71	712.17 ± 41.00	1,349.55 ± 87.33	63.24 ± 3.06
	F_(9,110)_	–	1.80	12.53	4.29
	*p*	–	0.102	0.0001	0.001

**Notes:**

WAP, Week after field planting.

The superscript asterisk (*) indicates significant differences of maize populations per treatment and *cv.Bhadra* (*p*-value < 0.05). The values correspond to the average of each parameter ± SE (*n* @ 3 WAP & 6 WAP = 20; *n* @ 10 WAP = 12).

**Figure 1:**
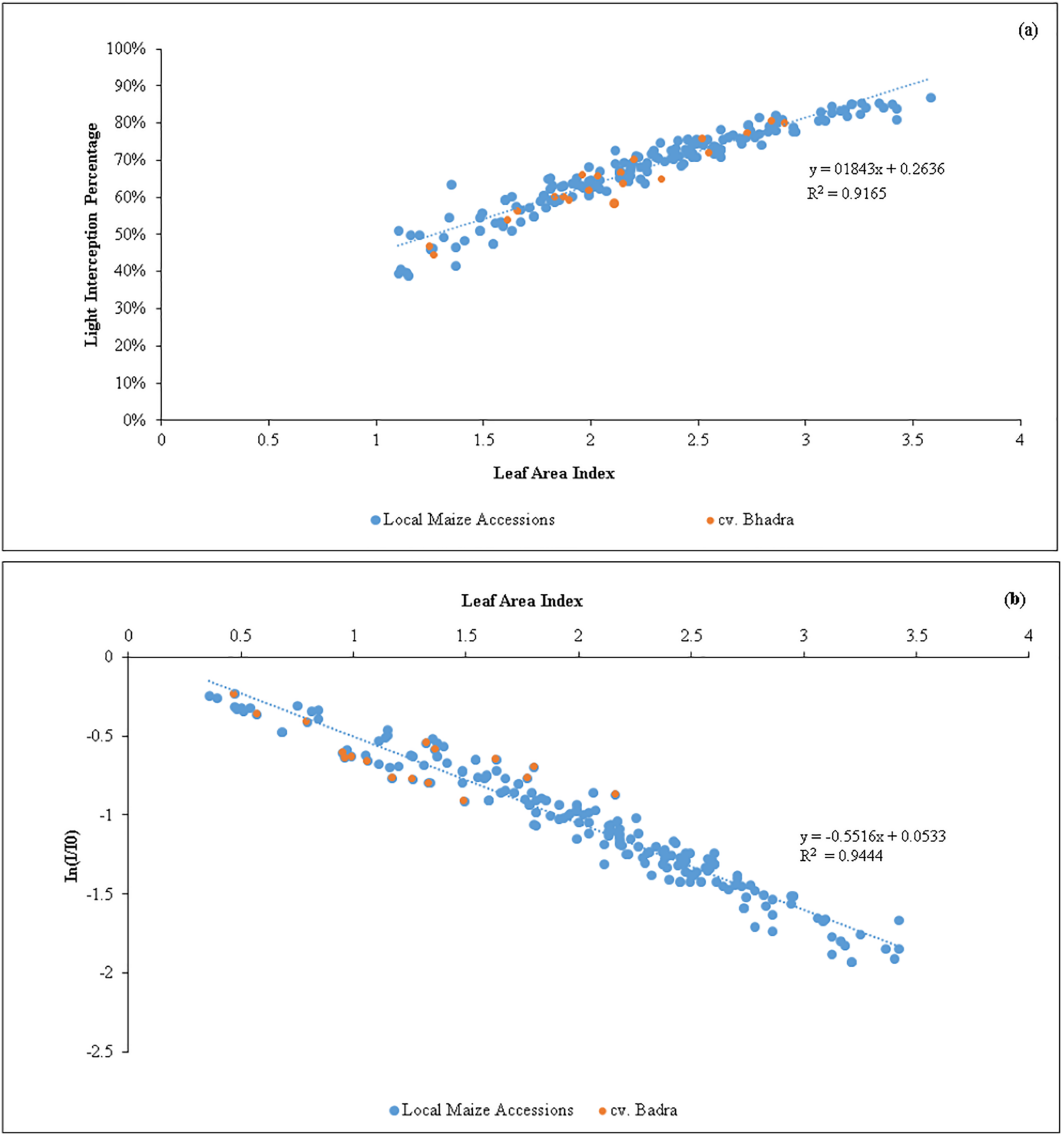
(A) Relationship between the variations of leaf area index (LAI) *vs* light interception percentage (LI %) of maize accessions (*n* = 200). (B) An inverse relationship between In (I/Io) *vs* leaf area index (LAI) of maize germplasm. The slope represents the light extinction coefficient (k) values (*n* = 200).

A strong positive regression (R^2^ = 0.92) between LAI and LI percent (Fig. 1) was found in the tested germplasm, showing that maize canopies with lower LAI exhibited lowered light interception percent. According to regression model, the maximum of 80% light interception can be obtained when the maize canopy reaches LAI = 3.0 to 3.5, and thereafter, producing LAI beyond 3.5 did not increase the light interception. Similar to this, there was a negative association between *In*(*I*/*I*_*0*_) and LAI (R^2^ = 0.95). We estimated the light extinction coefficient (*k*) of each tested germplasm at various growth stages using the slope of the regression line (Table 4). The mean *k*-values were shown non-significantly different, ranged from 0.33 in *SEU14* to 0.50 in *SEU9*, all maize germplasm showed an overall *k* of 0.5, indicating that the leaves produced were upright/eractophelic.

**Table 4:** Variations in light extinction coefficient (*k*) and fractional interception of solar radiation (*f*) at different stages of maize accessions.

Maize germplasms	Light extinction coefficient (*k*)					Light fractional interception (*f*)			
	3 WAP	6 WAP	10 WAP	Mean ± SE		3 WAP	6 WAP	10 WAP	Mean ± SE
*SEU2*	0.19	0.55	0.59	0.44 ± 0.13		0.25	0.78	0.67	0.57 ± 0.16
*SEU6*	0.26	0.56	0.42	0.41 ± 0.09		0.23	0.76	0.57	0.52 ± 0.15
*SEU9*	0.49	0.57	0.45	0.50 ± 0.04		0.47	0.80	0.45	0.57 ± 0.11
*SEU10*	0.32	0.61	0.52	0.48 ± 0.09		0.34	0.76	0.68	0.59 ± 0.13
*SEU14*	0.27	0.58	0.15	0.33 ± 0.13		0.23	0.74	0.23	0.40 ± 0.17
*SEU15*	0.30	0.65	0.21	0.39 ± 0.13		0.34	0.83	0.20	0.46 ± 0.19
*SEU16*	0.25	0.64	0.43	0.44 ± 0.11		0.27	0.74	0.59	0.53 ± 0.14
*SEU17*	0.18	0.58	0.31	0.36 ± 0.12		0.23	0.79	0.53	0.52 ± 0.16
*Pacific-999*	0.26	0.6	0.34	0.40 ± 0.10		0.21	0.82	0.62	0.55 ± 0.18
*cv.Bhadra*	0.42	0.62	0.31	0.45 ± 0.09		0.43	0.76	0.51	0.57 ± 0.10
F_(9,29)_	–	–	–	0.26		–	–	–	0.15
*p*	–	–	–	0.98		–	–	–	0.99

**Notes:**

WAP, Week after field planting.

The values correspond to the mean of each parameter ± SE (*n* = 20).

Additionally, the fractional light absorption (*f*) by the canopies of maize tends to vary depending on the stage of growth (Table 3). There were no significant differences in *f* between the local germplasms and the control plants, although values ranged from 0.4 in *SEU 14* to 0.59 in *SEU 10*. The *f* was lower at the three WAP, reached its peak at the six WAP for canopies, and then declined at the 10 WAP. The accession *SEU10* had the greatest value (*f* = 0.59), while *SEU2* and *SEU9* had similar values to *cv.Bhadra* (*f* = 0.57), the remaining treatments had values lower than 0.4, demonstrating that the maize accessions varied in their fractional interception of solar energy. The tested maize accessions did not differ significantly in terms of their chlorophyll contents (Table S1). In this case, nearly all of the accessions had chlorophyll levels in their leaves that were greater than >48.0 SPAD units.

### Chlorophyll fluorescence parameters

There were no appreciable differences in the mean maximum quantum yield by photosystem II (PSII) at light (*Q*_*y light*_: Fv/Fm) adapted leaf, which ranged between 0.64 to 0.68 (Table 5). A similar pattern was followed by *Qy*_*dark*_, with the only exception being the greater value of SEU6 and *Pacific-999* at six WAP.

**Table 5:** Variations in quantum yield in light and dark acclimated maize leaves across different stages.

Maize germplas ms	Quantum yield at the light acclimated leaf				Quantum yield at the dark acclimated leaf			
	3 WAP	6 WAP	10 WAP	Mean ± SE	3 WAP	6 WAP	10 WAP	Mean ± SE
*SEU2*	0.67 ± 0.01	0.63 ± 0.08	0.61 ± 0.01	0.63 ± 0.01	0.74 ± 0.01	0.73 ± 0.01	0.72 ± 0.01	0.73 ± 0.00
*SEU6*	0.68 ± 0.01	0.56 ± 0.08	0.63 ± 0.01	0.63 ± 0.01	0.73 ± 0.01	0.74 ± 0.01*	0.72 ± 0.01	0.73 ± 0.01
*SEU9*	0.66 ± 0.01	0.59 ± 0.08	0.60 ± 0.02	0.61 ± 0.01	0.72 ± 0.01	0.73 ± 0.01	0.72 ± 0.01	0.72 ± 0.00
*SEU10*	0.65 ± 0.01	0.54 ± 0.13	0.65 ± 0.01	0.61 ± 0.01	0.74 ± 0.01	0.69 ± 0.01	0.69 ± 0.01	0.70 ± 0.01
*SEU14*	0.64 ± 0.01	0.59 ± 0.07	0.58 ± 0.01	0.60 ± 0.01	0.72 ± 0.01	0.73 ± 0.01	0.67 ± 0.02	0.70 ± 0.01
*SEU15*	0.64 ± 0.01	0.60 ± 0.06	0.63 ± 0.01	0.62 ± 0.01	0.74 ± 0.01	0.71 ± 0.01	0.71 ± 0.01	0.72 ± 0.01
*SEU16*	0.65 ± 0.01	0.58 ± 0.09	0.60 ± 0.02	0.61 ± 0.01	0.73 ± 0.01	0.73 ± 0.00	0.70 ± 0.01	0.72 ± 0.00
*SEU17*	0.64 ± 0.01	0.61 ± 0.10	0.63 ± 0.01	0.63 ± 0.01	0.74 ± 0.01	0.72 ± 0.01	0.68 ± 0.01	0.69 ± 0.01
*Pacific-999*	0.66 ± 0.01	0.62 ± 0.07	0.60 ± 0.02	0.63 ± 0.01	0.73 ± 0.01	0.75 ± 0.01*	0.72 ± 0.01	0.73 ± 0.00
*cv.Bhadra*	0.67 ± 0.01	0.57 ± 0.09	0.61 ± 0.01	0.61 ± 0.01	0.73 ± 0.01	0.69 ± 0.01	0.70 ± 0.01	0.71 ± 0.01
F_(9,190)_	2.79	2.34	1.79	1.81	1.04	3.66	3.05	5.30
*p*	0.005	0.02	0.07	0.07	0.41	0.0001	0.002	0.0001

**Notes:**

WAP, Week after field planting.

* Indicates differences between the corresponding maize accession and *cv.Bhadra* (*p* < 0.05). The values correspond to the mean of each parameter ± SE (*n* = 20).

### Leaf gas exchange parameters

The photosynthetic leaf exchange parameters of maize plants were measured at six WAP (Table 6). The novel hybrid variety *Pacific-999* had denoted with significantly increased rate of photosynthesis A_N_ (30.45 μmol CO_2_ m^−2^s^−1^) in comparison to the control plant *cv.Bhadra* (23.2 μmol CO_2_ m^−2^s^−1^). Importantly, the native germplasm *SEU17*, *SEU9* and *SEU2* displayed significantly increased photosynthesis rates of 29.7, 28.9 and 26.2 (μmol CO_2_ m^−2^s^−1^) respectively than that of *cv. Bhadra*. It was possible to observe comparable variations in E, *Ci, and gs* among the studied maize germplasm.

**Table 6:** Variations in photosynthetic traits of maize accessions.

Maize germplasm	*A*_*N*_ (μmol m^−2^s^−1^)	*E* (mmol m^−2^s^−1^)	*Ci* (μmol mol^−1^)	*gs* (mmol m^−2^s^−1^)
*SEU2*	26.23 ± 0.74*	3.42 ± 0.30	97.52 ± 5.38	0.23 ± 0.03
*SEU6*	19.62 ± 0.37	2.45 ± 0.0	62.56 ± 1.70*	0.13 ± 0.00*
*SEU9*	28.93 ± 0.47*	3.44 ± 0.40	117.53 ± 4.98	0.26 ± 0.02
*SEU10*	24.41 ± 1.45	3.08 ± 0.28	82.16 ± 5.21	0.18 ± 0.02
*SEU14*	19.76 ± 1.97	2.76 ± 0.31	85.45 ± 13.57	0.15 ± 0.02
*SEU15*	22.33 ± 1.24	3.12 ± 0.18	81.32 ± 0.77	0.15 ± 0.02
*SEU16*	28.16 ± 1.58	3.73 ± 0.44	103.89 ± 5.95	0.28 ± 0.04
*SEU17*	29.74 ± 0.81*	3.98 ± 0.18	106.19 ± 3.31	0.33 ± 0.02
*Pacific-999*	30.45 ± 1.77*	3.95 ± 0.35	92.35 ± 3.55	0.29 ± 0.03
*Cv. Bhadra*	23.27 ± 0.47	3.62 ± 0.64	111.42 ± 12.5	0.28 ± 0.07
F_(9,87)_	11.91	2.53	5.77	9.96
p	0.0001	0.004	0.0001	0.0001

**Notes:**

*A*_*N*_, Photosynthetic rate at 410 ppm CO_2_ concentration; *E*, transpiration rate; *Ci*, intercellular CO_2_ concentration; *gs*, stomatal conductance.

* Indicates significant differences between tested maize populations and the *cv.Bhadra* (*p* < 0.05). The values correspond to the mean of each parameter ± SE (*n* = 16).

**Figure 2:**
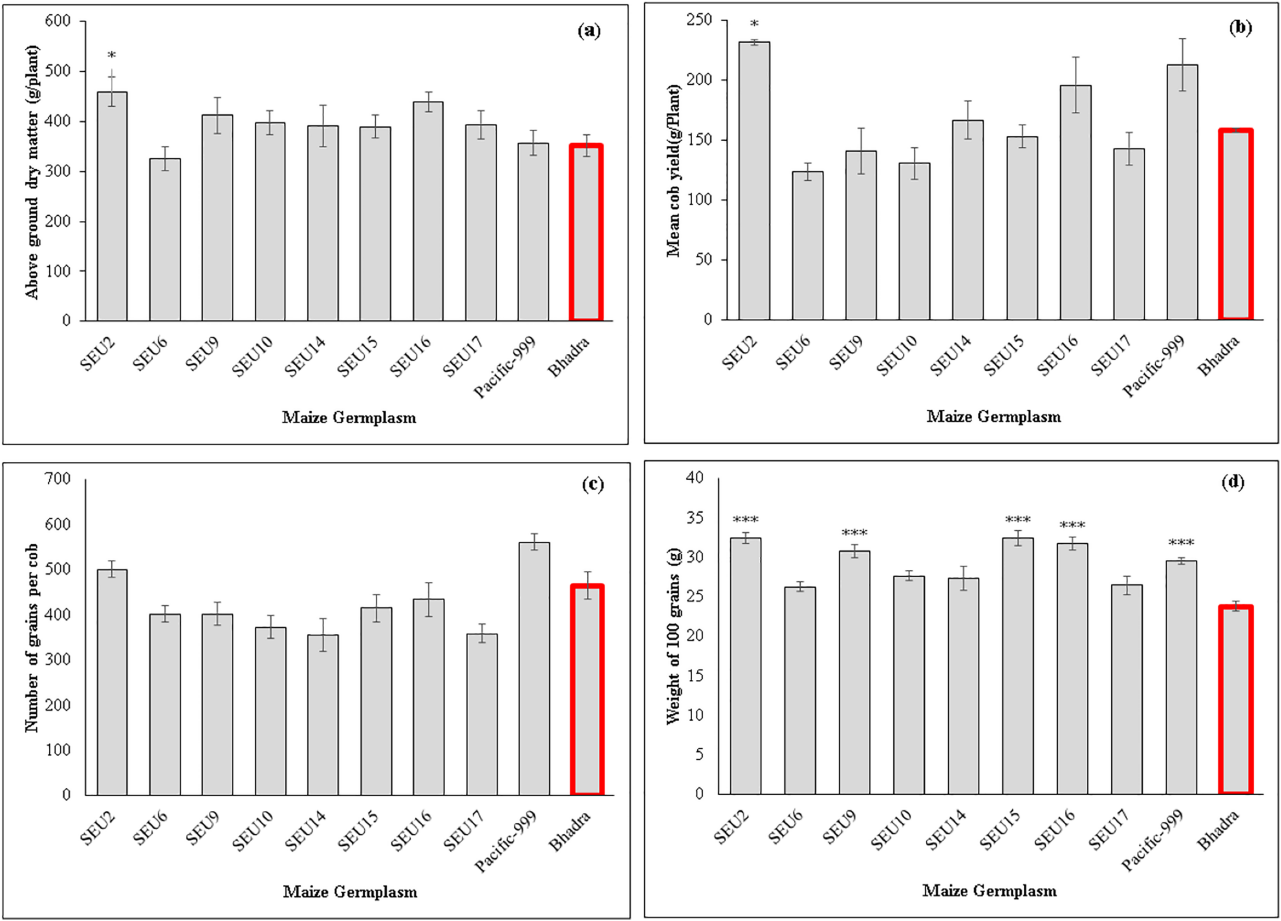
Above ground dry mass (A) cob yield per plant (B) number of grains per cob (C) 100 grain’s weight of maize germplasm. (D) The asterisk marks above the column (*, ***) represent the significant differences between corresponding germplasm and control (cv.Bhadra) at 5%, and 1%, significant level, respectively.

The control plants and variety *Pacific-999* showed increased stomatal conductance (0.29 mmol m^−2^s^−1^), while *SEU6* had significantly lowest than that of control variety. However, there were non-significant difference among other tested germplasm. Transpiration rates ranged between 2.45–3.98 mmol m^−2^s^−1^ among the tested germplasm and *SEU6* showed significantly lower than the control, whereas there was no significant difference among other tested germplasm Fig. 2 and Table 7 show the analysis of the biomass and yield characteristics of maize accessions. With regard to the yield components, *SEU2* produced significantly higher above-ground dry mass (AGDM) of 458.4 g Plant^−1^ than that of the control (290 g Plant^−1^) (Fig. 2A). The remaining germplasm typically had AGDM values that were comparable to the control plant. Similar trend was seen for cob yield per plant basis, *SEU2* produced the heaviest cobs (231.5 ± 2.2 g ) among the germplasm examined (Fig. 2B); which was significantly higher than the control (158.17 ± 1.63 g). Moreover, there were significantly higher 100 grain weight observed in *SEU2, 9, 15, 16*, and *Pacific-999* among them (Fig. 2D). The lengthiest cob was observed in *SEU2* closely followed by *SEU15*, *SEU6*, and *Pacific-999* were significantly longer than the the *cv.Bhadra*. The highest number of kernels were seen in a single row of cob in *Pacific-999* followed by *SEU2*.

**Table 7:** The cob characteristics of the maize germplasm at the physiological stage of maturity.

Maize germplasm	No of cobs per plant	Number of kernels per row (g)	Number of kernel rows per cob (g)	Cob length (cm)
*SEU2*	1.26 ± 0.13	38.25 ± 1.06	13.17 ± 0.53	24.64 ± 0.10*
*SEU6*	1.29 ± 0.20	31.25 ± 1.17	12.92 ± 0.47	20.06 ± 0.23*
*SEU9*	1.42 ± 0.04	30.58 ± 2.09	13.25 ± 0.48	18.71 ± 0.22*
*SEU10*	1.51 ± 0.20	30.83 ± 1.98	12.08 ± 0.38	19.96 ± 0.13*
*SEU14*	1.56 ± 0.16	27.42 ± 1.93	12.75 ± 0.25	18.90 ± 0.23*
*SEU15*	1.35 ± 0.07	29.73 ± 1.25	13.42 ± 0.67	22.36 ± 0.22*
*SEU16*	1.56 ± 0.11	32.17 ± 1.65	12.67 ± 0.50	18.76 ± 0.18*
*SEU17*	1.44 ± 0.19	27.83 ± 1.21	12.83 ± 0.51	17.75 ± 0.08*
*Pacific-999*	1.49 ± 0.12	40.83 ± 0.96*	13.67 ± 0.28	21.41 ± 0.17*
*cv.Bhadra*	1.23 ± 0.09	32.42 ± 1.55	14.25 ± 0.49	14.88 ± 0.13
F_(treatment, total)_	F_(9,39)_ = 0.75	F_(9,119)_ = 0.76	F_(9,120)_ = 1.63	F_(9,119)_ = 183.98
*p*	0.66	0.001	0.12	0.001

**Note:**

* Indicates significant differences within maize populations per treatment (*p* < 0.05). The values correspond to the mean of each parameter ± SE (*n* = 16).

Interesting characteristics of the maize accessions were revealed by the correlation matrix among the plant attributes. Overall, a significantly positive correlation between the percentage of PAR light interception (LI %), and A_N_ (0.21), AGDM (0.29) and weight of 100 kernels (0.34). Although the number of kernels developed in a cob (0.37) was shown significantly negative, indicating that the light penetration throughout the canopy is advantageous for the maize plant’s ability to synthesize photosynthates and engage carbohydrate utilization mechanisms. Additionally, significant correlation was evident between photosynthetic traits and grain yield characteristics in maize accessions, notably with respect to AGDM (0.59), cob weight (0.61), and kernel weight (0.385), respectively (Table S2).

## Discussion

Exploring the latent potentials of maize accessions through systematic plant screening techniques has enormous potential to identify candidate maize lines for future plant breeding programmes. In order to uncover the latent potentials of native Sri Lankan maize germplasm, the research presented here utilized cutting-edge technologies for determining photosynthetic features, plant canopy architecture, and biomass characteristics.

The maize germplasm was found to have strong variability in morphological and canopy architectural features, according to our findings, the *SEU2* and *SEU9* showed enhanced LAI and solar light interception percentage along with early blooming (44 d) characteristics. The LAI for maize was previously studied and found to be between 1.0 and 2.0 for the Konsur maize cultivar (Hokmalipour & Darbandi, 2012). LAI in the present study reached 3.0 at 6 WAP and showed a declining trend at 10 WAP, probably due to the senescence of older leaves as was reported by Rahman et al. (2010) studying the application of various nitrogen fertilizers to hybrid maize. As shown by Mangani et al. (2018), maize LAI can achieve a maximum of 3.5 to 4.6 during the mid-vegetative stage and tend to decrease at the grain filling stage in African maize varieties.

Our results showed that when the LAI reached values between 3.0 and 3.5, the maize canopy attained a maximum light interception percentage (80%). This can be seen as a novel discovery from the standpoint of plant breeding (Fig. 1). In order to maximize the solar light interception percentages, maize crops should be cultivated with canopies of LAI in the above range to optimize yield. Of the identified candidate lines, the native germplasm *SEU2*, *SEU9* and *SEU15* produced the required LAI (Table 2). As a result, plant breeders should concentrate on creating new maize varieties employing the native maize accessions that can reach an overall LAI of 3.0, as optimizing LAI tends to have a positive effect on biomass and grain yield production (Lei et al., 2010).

The *k* of a plant is theoretically connected to the leaf inclined angle (α) and solar zenith angle (θ). An elevated *k* value indicates that little radiation can penetrate the canopy’s understory, whereas a low *k* value indicates that high PAR may reach the bottom of the canopy. Typically, lower *k* values are found in crops with thin, upright leaves than in those with a more horizontally distributed leaf orientation (Azam-Ali & Squire, 2002). Based on the genotypes of plants’ physiological and morphological characteristics as well as their developmental stage, the *k* fluctuates. Our findings showed that, despite large variability at different plant growth stages, leaves of the tested maize accessions generally produce an upright stature (mean *k* < 0.4). Such large variations in *k* were also reported in rice (Mubarak et al., 2022), cassava (Adiele et al., 2021), wheat (Pradhan et al., 2018) and maize (Lacasa et al., 2021). The leaf chlorophyll content is critical in determining the amount of light absorbed and utilized in photosynthesis, which ultimately affects plant development and productivity (Taiz et al., 2015). However, our trial explicitly found that a sufficient amount of chlorophyll was presented in leaves at all stages of maize accession (>48.0 SPAD units) with minor variations. This corroborates previous works of Hokmalipour & Darbandi, 2012 as well as Mihai & Florin (2016) who reported an average of 50 SPAD units.

Concerning chlorophyll fluorescence, the Qy depicts the efficiency of PSII photochemistry (Zivcak, Olsovska & Marian, 2017) and found that the maximum *Qy*_*dark*_ was relatively smaller (0.47) value depending on temperature (25 to 30 °C). Our findings were slightly higher than the previous study as for *Qy*_*light*_ and *Qy*_*dark*_ remained above 0.67 and 0.70 respectively. This shows the native maize accessions are capable of performing high PSII chemistry to drive photosynthesis in leaves.

The leaf gas exchange characteristics are also thought to be one of the most reliable ways to evaluate the physiological performance of a crop at the leaf level. Theoretically, leaf stomatal conductance (*gs*) would result in an increase in photosynthetic rate. The bigger *gs* would also result in an increase in CO_2_ transport inside the leaf, and a simultaneous increase of water loss from the plant leaf cell. Photosynthetic characters may also vary due to plant genetic diversity in maize (Leitão et al., 2019), rice (Nagoor, 2013) and other cereal crops. Our findings suggested that *SEU2*, *SEU9* and *Pacific-999* exhibited significantly increased *A*_*N*_, while the former two germplasms exhibited moderately lower stomatal conductance and transpiration, suggesting that the native maize accessions have the capacity to succeed increased rates of photosynthesis (29.0 μmol CO_2_ m^−2^s^−1^) while minimizing water loss from the plants.

Recent research on maize cultivars in African fields revealed that A_N_ ranged between 23.00 and 25.00 μmol CO_2_ m^−2^s^−1^ and the *gs* ranged from 0.25 to 0.35 mmol m^−2^s^−1^ (Mangani et al., 2018). Additionally, Gujarat-Maize-6 (GM6) cultivar in India exhibits the same A_N_, *gs*, and *E* (Ramazan et al., 2021). Moreover, the *E* of the native pure maize line in Bangladesh is determined to be 3.5 mmol m^−2^s^−1^ at most, hence these results are consistent with those of the current investigation.

The performance of stomata is determined by developmental stage as well as environmental and genetic factors. Thus, the variation observed in previous studies is the result of soil water status, genetic variations and the developmental stage of the leaf analyzed (Prins et al., 2011). However, our improved photosynthesis in the identified germplasm is a result of the genetic nature of maize accessions as we kept the other field conditions and measured plant parameters constant (leaf age, soil moisture status). Further, the efficiency of CO_2_ fixation is also heavily dependent on the energy provided by the light reaction which could also be a limiting factor (Hirth et al., 2013).

Additionally, our findings showed that F_1_ hybrid variety *Pacific-999* expressed higher photosynthetic rates (A_N_) than the control plants *cv.Bhadra* (open pollinated variety), which may be attributable to the genotype’s hybrid vigour. To achieve such hybrid vigour in maize germplasms, the identified maize plant must first be let to undergo recurrent self-pollination, with those inbred lines (IL_5_), and then two-way maize crosses can be performed (Acquaah, 2012). Therefore, it is possible to generate more unique maize hybrid types by using native germplasm, particularly *SEU2* and *SEU9* to sustain increased biomass and grain production. According to Liao et al. (2022), the AGDM and grain yield of Chinese maize genotypes grown under control conditions are 300–400 g/plant and 210–220 g/plant, respectively. These findings support the conclusions of the current study.

Moreover, our findings indicated the significant correlation between photosynthetic properties of maize germplasm with the canopy architectural (LAI, *k*, and *f*) properties that determine the final biomass and grain yield production (Table 7). This situation can be explained from the standpoint of plant physiology; an increased light absorption caused by optimized leaf area and angle determined by *k* tend to absorb solar radiation at high rates because of optimized *f* by crop canopies.

This corresponds with previous work by Murchie, Pinto & Horton (2009), higher source-sink interactions in plants eventually boost grain output by translocating more photosynthates to cobs and other plant storage tissues. Furthermore, compared to late flowering plants, the early flowering characteristics of some maize accessions (44 d) are particularly advantageous in terms of solar radiation interception, leading to longer days for grain filling. Thus, the photosynthetic and gas exchange traits of the *SEU2* and *SEU9* maize genotypes showed improved AGDM and grain production as a result of the aforementioned superior canopy architecture.

## Conclusions

The purpose of this research was to identify efficient photosynthetic and biomass attributes of the maize accessions in order to propose novel, less resource-intensive cultivars to sustain global food security. The outcome of our study lead to the conclusion that maize accessions show large variations in the plant traits, and *SEU2* and *SEU9* consistently outperformed the elite cultivar *cv.Bhadra*. This observation has some profound implications for the maize breeding programme. First, once the inbred lines have been produced, the identified superior candidate genotypes can serve as a rich source of germplasm to identify novel hybrid maize crosses. Second, we report a novel observation, that the LAI threshold was between 3.0 to 3.5, which was sufficient to obtain 80% light interception, thereby ensuring the development of new cultivars with optimized canopies light penetration and improved radiation use efficiency. Third, the lower transpiration rates with improved photosynthesis will allow maize to be produced in water limited regions with promised grain yield production. Finally, the use of molecular tools is crucial, as genomic sequencing and marker-assisted selection will unveil the underlying genetic backgrounds of elite native maize germplasms.

## Supplemental Information

10.7717/peerj.15233/supp-1 Supplemental Information 1 Raw data for Table S1.

10.7717/peerj.15233/supp-2 Supplemental Information 2 Raw data for Table S2.

10.7717/peerj.15233/supp-3 Supplemental Information 3 Raw Data for Table 1.

10.7717/peerj.15233/supp-4 Supplemental Information 4 Raw data for Table 2.

10.7717/peerj.15233/supp-5 Supplemental Information 5 Raw data for Table 3.

10.7717/peerj.15233/supp-6 Supplemental Information 6 Raw data for Table 4.

10.7717/peerj.15233/supp-7 Supplemental Information 7 Raw data for Table 5.

10.7717/peerj.15233/supp-8 Supplemental Information 8 Raw Data for Table 6.

10.7717/peerj.15233/supp-9 Supplemental Information 9 Raw Data for Table 7.

10.7717/peerj.15233/supp-10 Supplemental Information 10 Chlorophyll content in young, fully expanded leaves at different stages of the maize germplasm. WAP: Week after field planting; The superscript * indicates differences between the corresponding maize landrace and *Bhadra* (*p*-value < 0.05). The values correspond to the average of each parameter ± SE (*n* = 20).

10.7717/peerj.15233/supp-11 Supplemental Information 11 Correlation analysis between leaf canopy architectural, photosynthetic and yield components of ten maize germplasm grown in optimum field conditions. The superscript *, **, ** indicates significant relationship at 10%, 5% and 1% significant level.

10.7717/peerj.15233/supp-12 Supplemental Information 12 Treatments and variety.

## Additional Information and Declarations

### Data Availability

The following information was supplied regarding data availability: The raw data is available in the Supplemental Files.
