# Supplementary material for: Will future maize improvement programs leverage the canopy light-interception, photosynthetic, and biomass capacities of traditional accessions?
Source: PeerJ. 2023 Apr 27;11:e15233. doi: 10.7717/peerj.15233 (PMC10149054; doi:10.7717/peerj.15233)
Supplement: Supplemental Information 10 — WAP: Week after field planting; The superscript * indicates differences between the corresponding maize landrace and Bhadra (p-value < 0.05). The values correspond to the average of each parameter ± SE (n = 20). [file peerj-11-15233-s010.docx]

| **Maize germplasms** | **3 WAP** | **6 WAP** | **10 WAP** | **Mean ± SE** |
| --- | --- | --- | --- | --- |
| *SEU2* | 48.54±1.11 | 54.61±1.13 | 52.37±1.40 | 51.24±0.89 |
| *SEU6* | 49.98±1.44 | 52.62±1.25 | 49.57±1.00* | 51.64±0.75 |
| *SEU9* | 47.52±1.61 | 53.17±1.05 | 54.40±1.59 | 51.78±1.15 |
| *SEU10* | 49.48±1.35 | 54.88±0.91 | 49.96±1.23 | 52.02±0.85 |
| *SEU14* | 45.33±1.20* | 53.63±1.09 | 55.12±1.35 | 52.35±0.85 |
| *SEU15* | 48.97±1.14 | 55.72±1.41 | 54.97±1.14 | 53.47±0.43 |
| *SEU16* | 48.03±1.78 | 52.13±1.23 | 55.84±1.30 | 52.00±0.85 |
| *SEU17* | 50.15±1.19 | 53.14±1.17 | 51.74±1.04 | 51.74±0.66 |
| *Pacific-999* | 46.93±1.19* | 53.62±1.21 | 55.61±1.26 | 52.33±0.79 |
| *cv.Bhadra* | 52.76±1.41 | 54.18±1.46 | 54.60±1.32 | 54.30±1.10 |
| F_(9,191)_ | 2.54 | 0.87 | 4.49 | 1.23 |
| p | 0.010 | 0.555 | 0.0001 | 0.832 |
| WAP: Week after field planting; The superscript * indicates differences between the corresponding maize landrace and *Bhadra* (p-value < 0.05). The values correspond to the average of each parameter ± SE (n=20). | | | | |

**Supplementary Table 1:** Chlorophyll content in young fully expanded leaf at different stages of maize germplasm.
