# Supplementary material for: Will future maize improvement programs leverage the canopy light-interception, photosynthetic, and biomass capacities of traditional accessions?
Source: PeerJ. 2023 Apr 27;11:e15233. doi: 10.7717/peerj.15233 (PMC10149054; doi:10.7717/peerj.15233)
Supplement: Supplemental Information 11 — The superscript *, **, ** indicates significant relationship at 10%, 5% and 1% significant level. [file peerj-11-15233-s011.docx]

**Supplementary Table 2:** Correlation analysis between the canopy architectural, photosynthetic, and yield components of the canopy of ten maize germplasm grown under optimal field conditions

|  | Below PAR | Amount of PAR attenuation | LAI | Qy_Light_ | Qy_Dark_ | Chlorophyll content | 100 Kernel’s weight | AGDM | Cob weight per plant | Number of Kernels per Cob | ***A_N_*** | ***E*** | ***C_i_*** | ***gs*** |
| --- | --- | --- | --- | --- | --- | --- | --- | --- | --- | --- | --- | --- | --- | --- |
| Below PAR | 1 |  |  |  |  |  |  |  |  |  |  |  |  |  |
| Amount of PAR attenuation | -0.151 | 1 |  |  |  |  |  |  |  |  |  |  |  |  |
| LAI | -0.028 | 0.003 | 1 |  |  |  |  |  |  |  |  |  |  |  |
| Qy_Light_ | 0.209 | 0.205 | -0.191 | 1 |  |  |  |  |  |  |  |  |  |  |
| Qy_Dark_ | 0.016 | 0.092 | -0.083 | 0.065 | 1 |  |  |  |  |  |  |  |  |  |
| Chlorophyll content | 0.143 | 0.026 | 0.037 | 0.013 | 0.003 | 1 |  |  |  |  |  |  |  |  |
| 100 Kernel’s weight | -0.392^**^ | 0.341^**^ | 0.102 | .262^*^ | 0.037 | 0.041 | 1 |  |  |  |  |  |  |  |
| AGDM | -0.378^**^ | 0.286^**^ | 0.083 | 0.278^*^ | 0.098 | 0.131 | 0.973^**^ | 1 |  |  |  |  |  |  |
| Cob weight per plant | 0.167 | 0.110 | 0.049 | 0.295 | 0.296 | -0.127 | 0.841^**^ | 0.846^**^ | 1 |  |  |  |  |  |
| Number of Kernels per Cob | -0.369^**^ | 0.257^*^ | 0.122 | 0.289^**^ | 0.142 | 0.056 | 0.995^**^ | 0.967^**^ | .816^**^ | 1 |  |  |  |  |
| ***A_N_*** | -0.276*^**^* | 0.211^*^ | 0.77 | 0.263^*^ | 0.179 | 0.021 | 0.385^**^ | 0.595^**^ | 0.651^**^ | 0.581^**^ | 1 |  |  |  |
| ***E*** | -0.283^**^ | 0.202 | 0.089 | 0.294^**^ | 0.141 | 0.127 | 0.670^**^ | 0.677^**^ | 0.509^**^ | 0.573^**^ | 0.901^**^ | 1 |  |  |
| ***Ci*** | 0.102 | 0.034 | -0.025 | 0.124 | 0.047 | 0.136 | 0.281^**^ | 0.291^**^ | -0.070 | 0.315^**^ | 0.492^**^ | 0.554^**^ | 1 |  |
| ***gs*** | 0.293^**^ | -0.218^*^ | -0.065 | 0.244^*^ | 0.067 | 0.126 | 0.471^**^ | 0.327^**^ | 0.529^**^ | 0.487^**^ | 0.898^**^ | 0.949^**^ | 0.660^**^ | 1 |
| The superscript *,**,** indicates significant relationship at 10%, 5% and 1% signifincat level | | | | | | | | | | | | | | |
