# Supplementary material for: Will future maize improvement programs leverage the canopy light-interception, photosynthetic, and biomass capacities of traditional accessions?
Source: PeerJ. 2023 Apr 27;11:e15233. doi: 10.7717/peerj.15233 (PMC10149054; doi:10.7717/peerj.15233)
Supplement: Supplemental Information 12 [file peerj-11-15233-s012.docx]

**Treatment, SEU Codes and Related Area Codes**

| **Treatment** | **SEU Code/ Variety** |
| --- | --- |
| 1 | SEU2 |
| 2 | SEU6 |
| 3 | SEU9 |
| 4 | SEU10 |
| 5 | SEU14 |
| 6 | SEU15 |
| 7 | SEU16 |
| 8 | SEU17 |
| 9 | CV.Pacific-999 |
| 10 | CV.Bhadra |
